# Artefact-free Evaluation of Metal Enhanced Fluorescence in Silica Coated Gold Nanoparticles

**DOI:** 10.1038/s41598-017-02678-0

**Published:** 2017-05-26

**Authors:** Tânia Ribeiro, Carlos Baleizão, José Paulo S. Farinha

**Affiliations:** Centro de Química-Física Molecular and Institute of Nanoscience and Nanotechnology, Instituto Superior Técnico - Universidade de Lisboa, Av. Rovisco Pais, 1049-001 Lisboa, Portugal

## Abstract

Metal nanoparticles can either quench or enhance the emission of dyes in their vicinity, but the precise measurement and understanding of this effect is still hindered by experimental artifacts, especially for particles in colloidal dispersion. Here, we introduce a new methodology to correct the inner filter effect of the metal on the dye emission. To test the method, we developed new hybrid nanoparticles with a gold core and a silica shell of precise thickness (tuned from 7 to 13 nm), with a high quantum yield perylenediimide dye on the surface. This novel approach effectively avoids fluorescence quenching, allowing us to measure emission enhancements of 5 to 30 times, with no change on the dye fluorescence lifetime. Being able to measure the emission enhancement in dye-metal hybrid nanoparticles in dispersion, free from inner filter and quenching artifacts, offers excellent prospects to guide the development of more efficient fluorescent probes, sensors and photonic devices.

## Introduction

Noble metal nanoparticles exhibit a localized surface plasmon resonance (LSPR) due to the interaction between incident light and surface electrons present in the metal conduction band. The LSPR depends on the particle material, shape and size, and can lead to large enhancements in the absorption and scattering of light^1–3^. Gold and silver nanoparticles are the most attractive for optical and biological applications, as their LSPR are located in the visible region of the spectrum. From these, gold nanoparticles (GNPs) are usually preferred because not only they have higher chemical stability and biocompatibility, but also their surface can be easily modified. This has led to many applications, including diagnostics, therapeutics, optical sensing and photovoltaics^4–7^.

The strong electromagnetic field generated at the surface of metal nanostructures upon resonant excitation has been reported to either quench^8–10^ or enhance^7, 11–18^ the photoluminescence of dyes in their vicinity. Generally, strong quenching is found for dye-metal separation distances around *ca*. 5 nm, with the enhancement effect being maximum at *ca*. 10 nm and decreasing rapidly with further increase in separation distance^19–22^. Metal enhanced fluorescence (MEF) has been related to both the increased excitation rate due to the enhancement of the local electrical field experienced by the dye^23, 24^, and the electromagnetic coupling of the dye with the metal nanoparticle, allowing the metal to partially transfer the excitation energy non-radiatively to the dyes, and also transmit the energy of the dye as radiation to the far field^25, 26^. This behavior results from the interplay of two opposite effects. On one hand, the electrical field enhancement decays exponentially from the metal nanoparticle surface outwards. On the other hand, quenching of the dye emission occurs up to 5 nm from the metal surface, an observation supported by simulation results with a polarizable continuum coupled quantum mechanical model that showed that the dye quantum yield is mostly suppressed up to 5 nm from the metal surface^27^.

Therefore, MEF depends critically on the distance between the dye and the metal surface, and on the spectral overlap of the metal LSPR with the emission or/and excitation spectra of the dye. Understanding and controlling these effects is thus essential for taking advantage of the plasmon-exciton interaction in new plasmonic devices^28^, and to improve fluorescence-based techniques such as single-molecule detection, bioimaging, DNA and protein analysis^19, 29–31^.

To control the strong distance dependence on metal-dye interactions, a variety of spacer materials, including silica^7, 10, 12, 13, 17, 18, 22, 32^, DNA^19, 33^ and polymers^11, 34, 35^ have been used. The disadvantage of using DNA and polymers as spacers is that due to their flexibility, these structures don’t allow a complete control of the metal-dye distance and can even fail to prevent dye-metal contact, leading to emission quenching. Contrarily, silica spacers with low porosity are rigid, allowing very good control of this distance, effectively avoiding dye-metal contact, and protecting the metal from ions present in biological media. Other advantages of using a silica layer are their robustness, chemical stability, and versatility of surface modification, for example, for the conjugation of biomolecules or dyes^36^. Although silica spacers from 3 to 90 nm have been described in the literature, there are contradictory results on which metal-dye distances lead to larger emission enhancements^7, 12, 13, 17, 18, 32^.

We believe that inconsistent enhancement results reported for colloidal systems with different metal-dye spacer materials are mostly due to experimental artifacts, such as dye quenching arising from contact with the metal when soft or porous spacers are used, light scattering by the particles, and/or inner filter effects related to light absorption by the metal nanoparticles.

**Figure 1:**
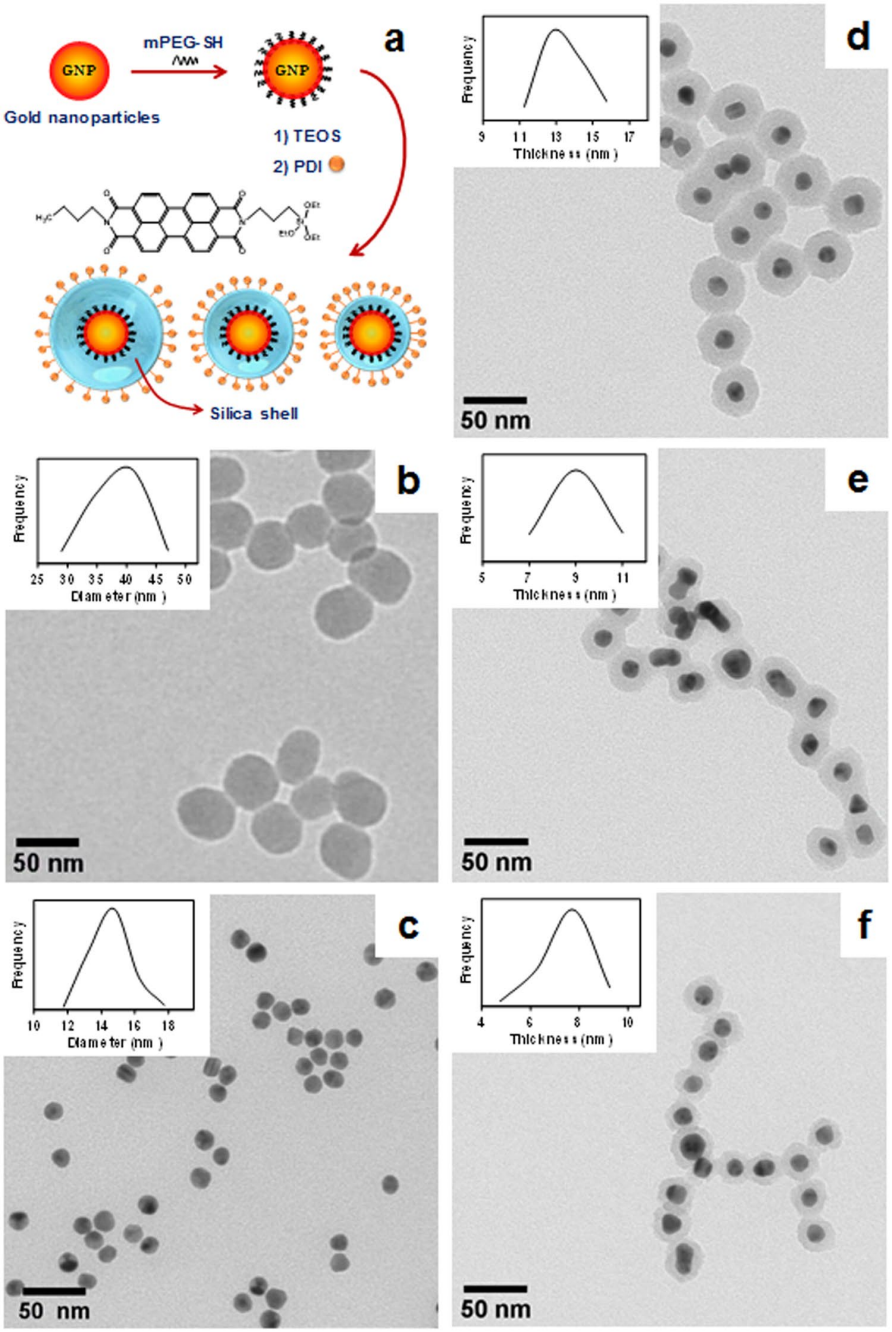
(**a**) GNPs were stabilized using a thiol-terminated poly(ethylene glycol), mPEG-SH, in order to transfer the GNPs to ethanol and grow a silica shell of controlled thickness. The surface of the core-shell nanoparticles was subsequently modified with a triethoxysilyl-modified perylene diimide (PDI). TEM images of SiNPs (**b**), GNPs (**c**), GNPSi13 (**d**), GNPSi9 (**e**), and GNPSi7 (**f**) show that the gold core is encapsulated in silica for all core-shell nanoparticles. The insets in (**b**,**c**) show the particle size distribution curve, and the insets in (**d**–**f**) show the silica thickness distribution curve.

To understand the relation between emission enhancement and metal-dye distance in colloidal systems, we prepared new hybrid metallic nanostructures consisting of a gold core and a silica shell of precisely controlled thickness, with a perylenediimide (PDI) derivative covalently attached to the silica outer surface (Fig. 1a). The PDI derivative (chosen to have absorption and emission spectra overlapping the LSPR of the GNPs for maximum enhancement effect)^37^, has very high fluorescence quantum yield, excellent photochemical stability, and an alkoxysilane moiety that reacts with the surface silanol groups of the silica shell^38^. This new architecture and the low porosity of the silica shell effectively prevent quenching of the dye fluorescence by the metal, while providing an excellent support for surface functionalization and thus guarantying that the dye-metal distance is equal to the shell thickness^39^. We studied the dependence of the photophysical properties of the PDI dye on the distance to the metal surface by varying the silica thickness around the distance range expected to have the strongest effect on the dye emission (*ca*. 10 nm)^11^. To determine the real enhancement in the dye emission intensity, it is necessary to avoid dye quenching by the metal and correct for the metal inner filter effect^40^. Here we propose a new methodology to correct the metal inner filter effect, based on the amount of light absorbed by the metal nanoparticle at the dye excitation and emission wavelengths^41–43^. We conclude that the largest emission enhancement is obtained for the shorter dye-GNP distance (7 nm), as predicted from the calculation of the electrical field enhancement around the metal nanoparticle. The effect is however larger than expected from the calculations, although the PDI fluorescence lifetime is not affected by the proximity of the gold.

The emission of dyes near plasmonic nanostructures is still not fully understood and characterized. A deeper knowledge of the real emission enhancement in colloidal hybrids of luminescent dyes and metal nanoparticles offers much better possibilities of harnessing the effect. By understanding the real magnitude of the emission enhancement and its dependence on the dye-metal distance, we hope to contribute to the development of more efficient probes, sensors and photonic devices such as organic solar cells.

## Results and Discussion

### Nanoparticle synthesis

Citrate-stabilized gold nanoparticles (GNPs) synthesized by the Turkevich method^44, 45^ were used as seeds to obtain monodisperse nanoparticles of uniform shape (Fig. 1c). Silica nanoparticles (SiNPs, Fig. 1b) were also synthesized to be used as reference. The gold nanoparticles were subsequently capped with a thiol end-labelled polyethylene glycol (mPEG-SH) in order to enhance the colloidal stability during their transfer from water to ethanol^46^, and centrifuged to separate the free citrate. The PEG coated gold nanoparticles (GNP-PEG) were then coated with a precisely controlled silica shell of thickness 13 nm (GNPSi13), 9 nm (GNPSi9) and 7 nm (GNPSi7), as shown in Fig. 1d–f. Particles with thinner silica shell showed irregular silica layer thickness, with areas of bare metal that lead to quenching of the dye emission. The values of the nanoparticle diameters (and silica shell thicknesses) were obtained by the analysis of several hundred particles in different TEM images (Table 1 and insets of Fig. 1b–f). Our minimum silica thickness is larger than 5 nm, thus avoiding quenching of the emission due to energy transfer from the dye to the metal^20, 27^. The porosity of the silica shell was evaluated by nitrogen adsorption experiments on the silica nanoparticles, with the pore volume (0.0017 cm^3^/g) being in the range expected for particles produced by the Stöber method^47^. Such low pore volume guaranties that the PDI is not able to diffuse across the silica shell during the reaction and contact the metal, a possibility when dyes are attached or adsorbed to metal particles encapsulated in mesoporous silica shells^22^.

**Table 1:** Average total particle diameter and silica shell thicknesses obtained by TEM for gold nanoparticles (GNP), silica-coated gold nanoparticles (GNPSi7, GNPSi9 and GNPSi13) and silica nanoparticles (SiNPs).

	Total diameter (nm)	Shell thickness (nm)
GNP	15 ± 1	—
GNPSi7	30 ± 3	7 ± 1
GNPSi9	33 ± 5	9 ± 1
GNPSi13	43 ± 6	13 ± 1
SiNPs	38 ± 4	—

**Figure 2:**
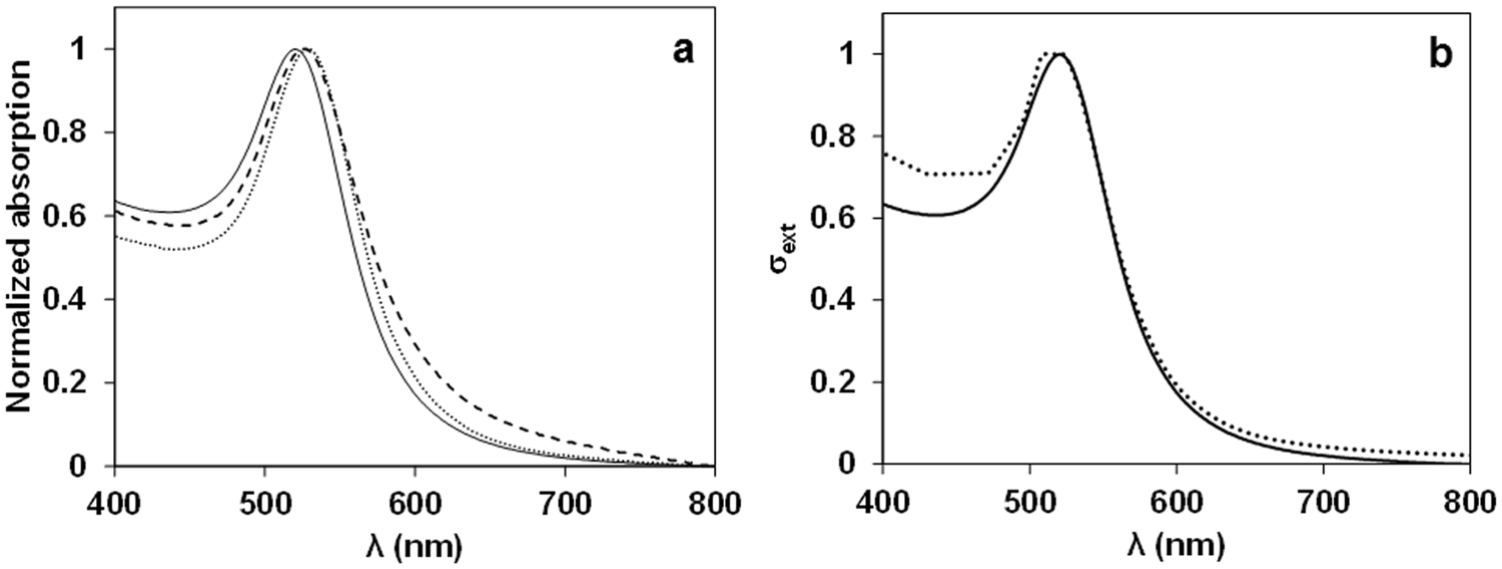
(**a**) Normalized absorption spectra of GNPs dispersed in water (solid curve), GNP-PEG (dashed curve) and GNPSi9 (dotted curve) dispersed in ethanol. A small red shift in the surface plasmon band of the gold is observed due to the higher refractive index of amorphous silica (1.46), compared to that of ethanol (1.36) and water (1.33). (**b**) The extinction spectra of GNPs dispersed in water (dotted curve) corresponds to the sum of the scattering and absorption contributions and can be calculated by the Mie theory (Supporting Information, section S1). The calculated extinction cross section (*σ*_*ext*_) agrees with that obtained experimentally for GNPs in water (solid curve).

The LSPR of the GNPs in water is located at 520 nm (Fig. 2a), showing a small red-shift to 526 nm upon surface modification with mPEG-SH (GNP-PEG) and transfer to ethanol, due to the higher refractive index of ethanol (1.36) compared to that of water (1.33). A further shift to 530 nm is observed after coating with silica (Fig. 2a), due to the higher refractive index of amorphous silica (1.46). Stability of nanoparticles at all preparation stages was confirmed by absorption measurements, with no aggregation being detected in the spectra (Fig. 2a). The experimental extinction spectra of GNPs dispersed in water (Fig. 2b) is in good agreement with that calculated as the sum of the scattering and absorption contributions using Mie theory (Supporting Information, section S1)^48^.

### PDI decorated gold-silica nanoparticles

**Figure 3:**
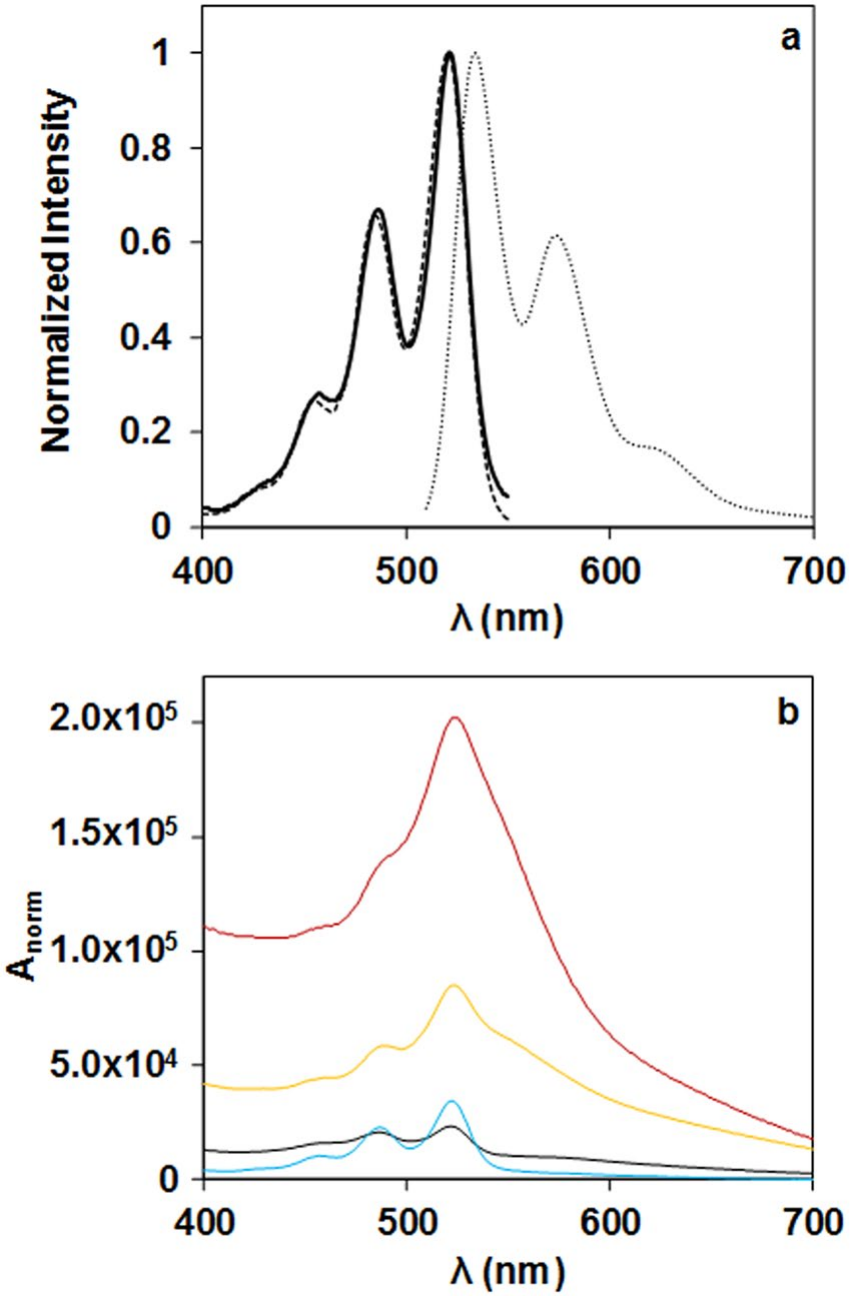
(**a**) Absorption (solid curve), excitation (λ_em_ = 580 nm, dashed curve) and emission (λ_ex_ = 480 nm, dotted curve) spectra of the PDI dye in ethanol; (**b**) Absorption spectra of SiNP-PDI (black curve), GNPSi13-PDI (blue curve), GNPSi9-PDI (yellow curve) and GNPSi7-PDI (red curve), normalized for equal PDI concentration.

The surface of the silica-coated gold nanoparticles GNPSi7, GNPSi9 and GNPSi13 were decorated with a controlled amount of PDI, determined by separating the unreacted PDI by centrifugation, and measuring the absorption spectra of the PDI-labelled nanoparticles in ethanol. For the PDI-labelled SiNPs, the spectrum corresponds to the sum of the SiNPs scattering and the absorption of surface-attached PDI. We correct for light scattering, by subtracting the spectra of unlabelled SiNPs of the same size (which corresponds to the inverse fourth power of the wavelength due to scattering) matched for the same scattering intensity (Fig. S9 - Supporting Information), and calculated the PDI concentration using the molar extinction coefficient of PDI in ethanol at 22 °C (${\varepsilon }_{22}^{521nm}$ = 15895 cm^−1^ M^−1^). Using the exact amount of SiNPs and their diameter obtained by TEM, we calculated the surface density of PDI, which was used in all core-shell nanoparticles. The absorption, excitation and emission spectra of PDI in ethanol are presented in Fig. 3a.

When the absorption spectra of the nanoparticles surface modified with PDI (GNPSi7-PDI, GNPSi9-PDI and GNPSi13-PDI) were normalized to the same PDI concentration, we observed an increase in the normalized absorbance (A/[PDI]) with the decrease in the shell thickness of the core-shell nanoparticles, which are always larger than for SiNP-PDI (Fig. 3b). This is due to the contribution of the gold LSPR at 530 nm overlapping the absorption maximum of PDI.

In order to study the effect of the metal nanoparticle on dye emission, we selected our PDI derivative so that its absorption overlaps the LSPR of the GNPs. However, in the overlapped spectral region the excitation light is distributed between the GNPs and the dyes, so that to calculate the effective excitation intensity of the PDI, we have to correct for the fraction of light that is absorbed by the GNPs. Furthermore, the broad absorption of the GNPs (extending beyond 600 nm) also overlaps the emission of PDI and therefore the light emitted by the dye can also be re-absorbed by the GNPs. These artefacts, usually known as inner filter effects, prevent the measurement of the real dye emission efficiency. Inner filter effects can be corrected by considering the fraction of excitation light that is absorbed by the dye, and the fraction of the light emitted by the dye that is re-absorbed by the GNPs at the wavelength used to detect the emission^41–43^

1 $$F{I}_{GNPSi{\mathbb{\mbox{--}}}PDI}=F{I}_{GNPSi{\mathbb{\mbox{--}}}PDI}^{0}\times \frac{{\varepsilon }_{SiNP{\mathbb{\mbox{--}}}PDI}^{\lambda ex}[{\rm{PDI}}]+{\varepsilon }_{Au}^{\lambda ex}\,[\text{Au}]+{\varepsilon }_{Au}^{\lambda em}\,[\text{Au}]}{{\varepsilon }_{SiNP{\mathbb{\mbox{--}}}PDI}^{\lambda ex}\,[{\rm{PDI}}]}$$

where $F{I}_{GNPSi{\mathbb{\mbox{--}}}PDI}^{0}$ is the measured emission fluorescence, ${\varepsilon }_{SiNP{\mathbb{\mbox{--}}}PDI}^{\lambda ex}\,[\text{PDI}]$ is the optical density of SiNP-PDI at the excitation wavelength *λ*_*ex*_ and dye concentration [PDI], ${\varepsilon }_{Au}^{\lambda ex}\,[\text{Au}]$ is the optical density of GNPs at the same excitation wavelength, and ${\varepsilon }_{Au}^{\lambda em}\,[\text{Au}]$ is the optical density of GNP at the wavelength *λ*_*em*_ used to measure the emission intensity.

**Figure 4:**
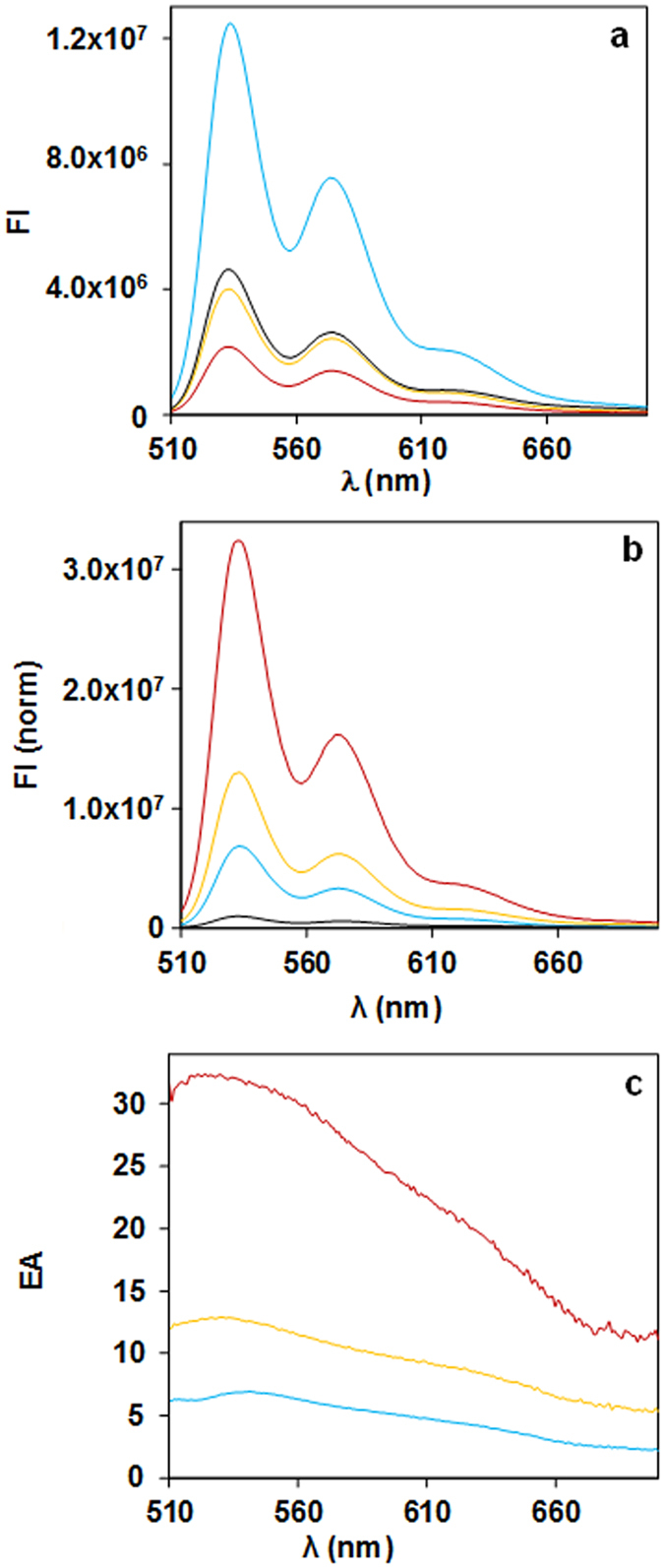
Emission spectra (*λ*_*ex*_ = 480 nm, right-angle acquisition in ethanol) normalized for the same PDI concentration, [PDI] = 10^−6^ M, obtained for SiNP-PDI (black curve), GNPSi13-PDI (blue curve), GNPSi9-PDI (yellow curve) and GNPSi7-PDI (red curve), before (**a**) and after (**b**) correction of the inner filter effects with equation (1). The corresponding emission intensity amplification curves calculated with equation (2), (**c**), show maxima around the GNPs LSPR.

In Fig. 4, we show the emission spectra of all samples using excitation light of 480 nm, measured in right-angle geometry (results obtained in front-face geometry and also at half the particle concentration are shown in Supporting Information, Figs S1–S3). The emission spectra of SiNP-PDI and GNPSi-PDI are shown in Fig. 4a after normalization to exactly the same PDI concentration. The emission by the particles with thinner silica shell (GNPSi9-PDI and GNPSi7-PDI) is lower than that of the silica particles SiNP-PDI, while that of GNPSi13-PDI is larger. This is because all samples were prepared with the same number of dyes/nm^2^, and therefore the particles with the thicker silica shell (larger surface area) have a larger amount of PDI/particle, which means that the ratio of gold to dye absorption is lower and the inner filter effect by the gold nanoparticle is less important. This effect masks the emission enhancement of the dyes so that, apparently, the particles with thinner shell show fluorescence quenching (lower emission intensity than the SiNP-PDI), while the particles with thicker silica shell show enhancement. In order to obtain the real emission enhancement, it is thus necessary to correct for the gold inner filter effect. Indeed, after correction with equation (1), the emission spectra of the GNPSi-PDI dispersions (Fig. 4b) show a clear increase in the emission intensity as the silica shell thickness decreases. This represents the real enhancement in PDI emission due to the proximity to the gold core, which increases as the silica shell thickness decreases. In order to quantify this emission enhancement, we calculated the enhancement amplification factor *EA* by dividing the emission intensity for each GNPSi-PDI sample by the intensity of the (gold-free) SiNP-PDI at each emission wavelength.

2 $$EA=\frac{F{I}_{GNPSi{\mathbb{\mbox{--}}}PDI}^{\lambda em}}{F{I}_{SiNP{\mathbb{\mbox{--}}}PDI}^{\lambda em}}$$

The emission amplification for the different GNPSi-PDI samples as a function of emission wavelength is shown in Fig. 4c (this is for spectra measured at right-angle geometry, for the spectra measured in front-face geometry and for the diluted samples see Fig. S4 in Supporting Information). The maximum emission amplification *EA*_*max*_ values are obtained at the wavelength of the GNPs LSPR, with average values of 6, 12 and 31, for silica shell thickness decreasing from 13 to 9 and 7 nm (samples GNPSi13-PDI, GNPSi9-PDI and GNPSi7-PDI, respectively). These results do not depend significantly on the acquiring geometry (right-angle or front-face) and nanoparticle concentration (Table S1 Supporting Information).

**Figure 5:**
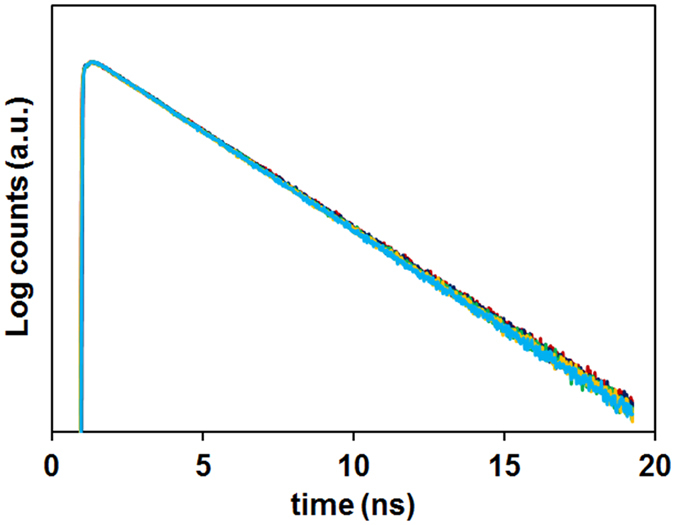
Luminescence intensity decay curves of PDI (red), GNPSi13-PDI (green), GNPSi9-PDI (yellow), GNPSi7-PDI (cyan) and SiNP-PDI (blue) nanoparticles using λ_exc_ = 460 nm, with emission observed at 530 nm.

One mechanism that can lead to emission enhancement is the increase in the radiative decay rate *k*_*r*_ of the dye when close to the gold nanoparticle^49^. The emission dipole of the dye induces a macroscopic polarization in the metal nanoparticle (larger near the frequency of the electronic plasmon resonance). This response increases the total emission dipole, resulting in an increase in the radiative emission rate^50^. However, since we use a PDI dye with very high fluorescence quantum yield (*ca.* 0.85)^38^, any change in *k*_*r*_ would be reflected in its fluorescence lifetime *τ* ^51^. Surprisingly, the fluorescence intensity decay curves of PDI and of GNPSi-PDI and SiNP-PDI in ethanol, measured at 530 nm with 460 nm excitation (Fig. 5), can all be fitted to a single exponential function with the same fluorescence decay lifetime, *τ* = 4.1 ns (Table S2, Supporting Information). This shows that the presence of the gold core does not influence the dye’s internal quantum structure and the radiative decay rate is not changed. We thus conclude that in our system the metal nanoparticle is only increasing the intensity of the local electric field at the dye position, thus enhancing the dye excitation and therefore its photoluminescence intensity^52^.

**Figure 6:**
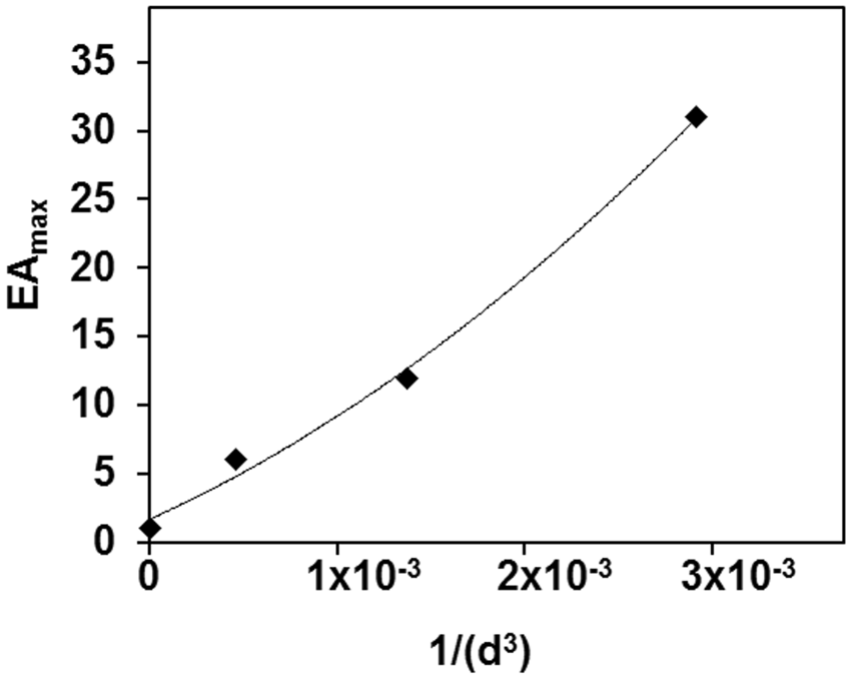
Representation of the experimental values of *EA*_*max*_ fitted with equation (3).

To verify this hypothesis, we calculated the intensity of the electric field at the silica surface using a discrete dipolar approximation (Supporting Information, section S2)^53^. The relation between the emission amplification *EA* and the separation distance *d* between the PDI molecules and the gold surface was determined considering that the enhancement in the field intensity exciting the PDI dyes at the silica surface, (*E*_*PDI*_/*E*_*inc*_)^2^, is directly reflected on the dye emission efficiency (due to its high fluorescence quantum yield). In this case, the emission amplification can be calculated as *EA*′ = (*E*_*PDI*_/*E*_*inc*_)^2^ so that

3 $$EA^{\prime} =1+\frac{2A}{{d}^{3}}+\frac{{A}^{2}}{{d}^{6}}$$

4 $$A=2{r}_{Au}^{3}(\frac{{\varepsilon }_{Au}-{\varepsilon }_{m}}{{\varepsilon }_{Au}+2{\varepsilon }_{m}})$$

where *ε*_*Au*_ is the dielectric function of gold and *ε*_*m*_ is the dielectric constant of the medium. Using the values of the dielectric functions of gold determined for the calculation of the extinction spectrum of GNPs (Supporting Information, section S1) at the excitation wavelength of 480 nm, considering *ε*_*m*_ = 1.99 (the dielectric constant of a mixture of silica, *n* = 1.46, and ethanol, *n* = 1.36), and the GNPs radius *r*_*Au*_ = 7.5 nm, we calculated *A* = (9.9 nm)^3^. The emission amplification decays sharply with the dye-metal distance (Fig. S5 in Supporting Information), with large metal-dye separations (1/*d* = 0) resulting in an enhancement of 1 as expected. In Fig. 6 we represent experimental *EA*_*max*_ data as a function of 1/*d* ^3^, fitting our data with equation (3) to yield *A* = (11.5 nm)^3^. This value is quite close to the calculated value, *A* = (9.9 nm)^3^, considering the approximations in the model. The calculated *A* value corresponds to maximum enhancement values of $E{A}_{{\max }}^{^{\prime} }$ of 2, 4 and 12 for dye-metal separation distances of 13, 9 and 7 nm, respectively. These values are *ca*. 3 times lower than those obtained experimentally (6, 12 and 31, respectively), but closely follow the obtained increase in absorption (Fig. 3b). This indicates that although the simple discrete dipolar approximation model is able to predict the increase in absorption for closer dye-metal distances, it does not completely reflect the emission enhancement values obtained. One possible reason for this mismatch between the calculated and experimental enhancement values is the dipolar approximation used in the calculations, ignoring quadrupolar modes and higher order multipoles^3^. However, this approximation is not likely to account for the difference observed, so that there is probably also an effect of the metal nanoparticle on the dye emission. This is intriguing since no alteration of the PDI emission lifetime was observed. Re-radiation of the dye emission by metal nanoparticles has been proved to be a robust and long-range effect, due to the larger oscillator strength of the nanoparticles relative to the dye^54^, but it remains to be determined if the nano-antennae can radiate the dye emission without affecting its quantum structure. Another possible effect is the cooperative emission by the ensemble of dyes located at the silica surface, for which strong mixing of super-radiant and sub-radiant states is expected from a certain dye-metal distance, resulting in possible further enhancement which decays with the increase of their separation distance^55^.

**Figure 7:**
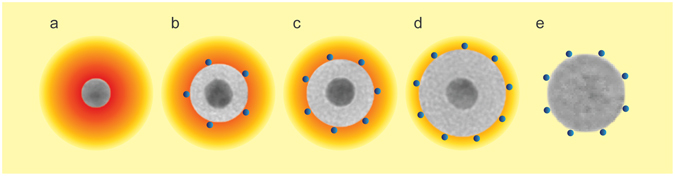
Cartoon of the field amplification around a GNP (**a**). PDI dyes on the surface of the gold-silica core-shell nanoparticles will feel a lower field amplification as the silica shell gets thicker ((**b**) GNPSi7-PDI; (**c**) GNPSi9-PDI; (**d**) GNPSi13-PDI). Dyes on the surface of SiNP-PDI, used as reference, are not subject to field enhancement (**e**).

Our results are consistent with a mechanism in which the proximal GNPs serve as antennae for the incoming excitation light, with the metal particles enhancing the electromagnetic field when excited at their LSPR, as a result of the polarization associated with the collective electron oscillation in the particles. When the GNPSi-PDI samples are irradiated, the PDI dyes on their surface will be subjected to a progressively stronger excitation field as the silica shell gets thinner (cartoon in Fig. 7). However, the effect cannot be completely predicted by our field enhancement calculations. Another mechanism can be contributing to the emission enhancement, that does not change the fluorescence lifetime of the dye and is not apparent in other dye-metal colloidal systems, probably due to masking by quenching and inner filter effects. Appropriate correction of inner filter effects and use of a spacer that effectively prevents dye-metal contact are key factors to study emission enhancement in colloidal systems. Use of the plasmon-exciton interaction in colloidal systems would strongly profit from avoiding the inner filter effect described here. To this end, we plan to either use a dye with a large Stökes shift (in which the absorption overlaps the LSPR of the GNPs but the emission falls on a region where there is no absorption of gold) or use a Förster resonance energy transfer (FRET) pair, where donor and acceptor molecules are located at precise distances from the gold core, with the donor absorption overlapping the LSPR of the GNPs, and the emission of the acceptor falling on a region where there is no GNPs absorption.

### Road map for artefact-free measurement of emission enhancement

The method we propose can be used to measure artefact-free emission enhancement in systems using different fluorophores and plasmonic nanoparticles. Here, we describe a road map for application of this method to different dye-metal conjugates.

In addition to a dispersion of the fluorophore-labeled plasmonic nanoparticles (Sample GNPSi-PDI), the method requires the use of three reference nanoparticle dispersions, one of the plasmonic nanoparticles without fluorophore (Sample GNPSi), one of nanoparticles with the same surface composition, containing the fluorophores but no metal (Sample SiNP-PDI), and another of nanoparticles with the same surface composition, but without fluorophores or metal (Sample SiNP).

#### Nanoparticle concentration

The concentration of the plasmonic nanoparticles is either calculated from their absorbance spectra, or estimated from the amount of metal salt used in the synthesis.

#### Amount of fluorophore in the nanoparticles

This is determined using the reference samples SiNP-PDI and SiNP. The nanoparticles in sample SiNP-PDI are prepared with exactly the same amount of fluorophore used on the plasmonic nanoparticles. Dispersions of SiNP-PDI and SiNP were prepared by weighting a determined amount of the dried nanoparticles, which were dispersed in a known volume of solvent. The UV-vis absorption spectrum of sample SiNP-PDI corresponds to the sum of two components: the scattering by the nanoparticles and the absorption of the fluorophore. The UV-vis absorption spectrum of sample SiNP corresponds only to the light scattering component. By subtracting the spectra of SiNP from that of SiNP-PDI (normalizing the spectra using the wavelength range where the dye does not absorb, to correct for eventual minor baseline mismatches), we obtain the absorption spectra of the fluorophore in the nanoparticles, corrected for nanoparticle scattering. From this spectrum, we can calculate the fluorophore concentration using its molar extinction coefficient, and from this value obtain the surface density of the fluorophore. This value is then used to calculate the amount of fluorophores in all samples.

#### Emission spectra correction

The emission spectra of the different GNPSi-PDI samples with known nanoparticle and fluorophore concentrations is measured in either right angle or front face geometry (using the same geometry for all samples). Correction of the emission spectra intensity $F{I}_{GNPSi{\mathbb{\mbox{--}}}PDI}^{0}$ for the inner filter effect is done with equation (1). If the UV-vis absorption spectra are measured at (or normalized to) the same concentration of fluorophores and metal nanoparticles, equation (1) can be represented as

5 $$F{I}_{GNPSi{\mathbb{\mbox{--}}}PDI}({\lambda }_{em})=F{I}_{GNPSi{\mathbb{\mbox{--}}}PDI}^{0}({\lambda }_{em})\times \frac{{A}_{SiNP{\mathbb{\mbox{--}}}PDI}({\lambda }_{ex})+{A}_{GNPSi}({\lambda }_{ex})+{A}_{GNPSi}({\lambda }_{em})}{{A}_{SiNP{\mathbb{\mbox{--}}}PDI}({\lambda }_{ex})}$$

where *A*_*SiNP*–*PDI*_ (*λ*_*ex*_) is the optical density of the dye-labelled nanoparticle dispersion (sample SiNP-PDI) at the excitation wavelength used to measure the emission spectra of the GNPSi-PDI sample (*λ*_*ex*_), obtained from the corresponding UV-vis absorption spectra; *A*_*GNPSi*_ (*λ*_*ex*_) is the optical density of the metal nanoparticles with no fluorophore (sample GNPSi) at the excitation wavelength used to measure the emission spectra of the GNPSi-PDI sample (*λ*_*ex*_), obtained from the corresponding UV-vis absorption spectra; and *A*_*GNPSi*_ (*λ*_*em*_) is the optical density of the metal nanoparticles with no fluorophore (sample GNPSi) at the wavelength of emission (*λ*_*em*_) of the GNPSi-PDI emission spectra, obtained from the corresponding UV-vis absorption spectra.

## Conclusions

We prepared new hybrid core-shell gold-silica nanoparticles with PDI fluorescent dyes attached to the silica surface at a precisely controlled distance from the gold core. The proposed correction method for the inner filter effect of the gold allow us to predict the real enhancement in the emission of the dye in the proximity of the GNP. The emission enhancement was found to occur without change in the dye emission lifetime, so it has been attributed to the increased local electromagnetic field around the metal. The dependence on the dye-metal distance compares well to that calculated using a discrete dipole approximation, however the later account for only part of the observed enhancement. To our knowledge, this is the first time that the fluorescence enhancement in hybrid nanoparticles labeled with a high quantum yield dye was measured without the influence of fluorescence quenching and inner filter effects. Since most metal enhanced fluorescence (MEF), surface-enhanced Raman spectroscopy (SERS) and metal enhanced Förster resonance energy transfer (ME-FRET) experiments of nanoparticles in dispersion described in the literature are affected by inner filter effects, the reported enhancements are probably under-evaluated and therefore, by using the proposed correction method the enhancement effect would be larger, closer to the values obtained in experiments on surfaces (in the case of MEF and ME-FRET, if no other artefacts are present, such as emission quenching due to poor isolation of the dyes from the metal). Besides its importance to understanding the enhancement effects in colloidal assemblies of dyes and metal nanoparticles, our results also open possible routes for application in the performance increase of probes, nanosensors and photonic devices such as organic photovoltaics.

## Methods

### Materials

Gold (III) chloride trihydrate (HAuCl_4_·3H_2_O, Sigma-Aldrich, 99.9+% metal basis), sodium citrate dihydrate (Na_3_C_6_H_5_O_7_·2H_2_O, SAFC, ≥99%), ethanol (Panreac, 99.5% PA), O-[2-(3-mercaptopropionylamino)ethyl]-O’-methylpolyethylene glycol (mPEG-SH, *M*_*w*_ 5000, Sigma-Aldrich), ammonium hydroxide solution 25% (Fluka) and tetraethyl orthosilicate (TEOS, Aldrich, 98%) were used as received. Deionized water from a Millipore system Milli-Q ≥ 18 MΩcm was used in the synthesis of nanoparticles and in the capping of GNP with mPEG-SH. The perylenediimide derivative (PDI) (Fig. 1a) was prepared as previously described^38, 39^.

### Methods

*Gold nanoparticles (GNPs) synthesis*Monodisperse gold nanoparticles (GNPs), 15 nm in diameter, were synthesized in two steps, using the Turkevich method^44, 45^, involving reduction and stabilization of gold chloride (HAuCl_4_) by citrate. The first step was the synthesis of gold seeds. Briefly, an aqueous solution of sodium citrate (38.8 mM, 5 mL) was rapidly added into a boiling aqueous solution of HAuCl_4_ (1 mM, 50 mL) under vigorous magnetic stirring. After boiling during 15 minutes, the solution was removed from the bath and cooled to room temperature. These particles were used as seeds to obtain monodisperse GNPs (Fig. 1c). A freshly prepared aqueous solution of HAuCl_4_ (0.296 mM, 125 mL) was heated to boiling. Next, a previously sonicated solution of GNP seeds (1.157 g) and an aqueous solution of sodium citrate (38.8 mM, 4.68 g) were added sequentially under vigorous magnetic stirring. After boiling 30 minutes, an additional solution of sodium citrate (38.8 mM, 5.0 g) with a molar proportion of 5:1 citrate:Au was added to the boiling solution and the reaction proceeded for one hour more.

#### *Silica nanoparticles (SiNPs) synthesis*

Silica nanoparticles (SiNPs) were synthesized by the hydrolysis/condensation of TEOS with aqueous ammonia, according to the reported Stöber method^56^. The molar ratios for the SiNPs were 1:1:22:94 of NH_3_:TEOS:H_2_O:ethanol.

#### *Capping of GNPs with mPEG-SH*

The GNPs were capped with mPEG-SH (using 4 molecules/nm^2^ of GNP surface)^46^. An aqueous solution of mPEG-SH (1 × 10^−4^ M) previously sonicated was added dropwise with vigorous magnetic stirring to GNPs aqueous dispersion. The reaction proceeded during 30 minutes and the dispersion was centrifuged twice at 3000 rpm during 6 h each cycle. The GNP-PEG were re-dispersed in absolute ethanol.

#### *Silica coated gold nanoparticles synthesis (GNPSi)*

The synthesis of a silica shell on the GNPs was performed by a modified Stöber method^56^, using 124, 49 and 25 μL of a 0.1 M ethanol solution of the precursor (TEOS), to obtain silica shell thicknesses of 13, 9 and 7 nm (for GNPSi13, GNPSi9 and GNPSi7). Milli-Q water (7.62 M) and the ammonium hydroxide solution (0.14 M) were added to the previously sonicated dispersion of GNP-PEG (1.81 mM, 0.90 mM and 0.36 mM of gold atoms for GNPSi7, GNPSi9 and GNPSi13) in absolute ethanol at 30 °C under magnetic stirring. The ethanol concentration in the reactions was 14.5 M. The final dispersion was centrifuged twice at 1000 rpm during 3 h each cycle. The particles were re-dispersed in absolute ethanol. TEM Images of the core-shell nanoparticles are presented in Figs S6–S8 (Supporting Information) with the corresponding diameter distribution.

#### *Surface modification of silica and GNPSi nanoparticles with PDI dye*

A solution of PDI in ethanol (1.33 × 10^−5^ M) was added to the dispersion of GNPSi or SiNPs, under magnetic stirring at 30 °C. The proportion of PDI used in the reactions was 4 *μ*mol/m^2^ of nanoparticles surface. The reaction proceeded for 48 h. The nanoparticles were centrifuged twice at 1000 rpm during 3 hours each cycle to remove the dye molecules that didn’t react. The nanoparticles were re-dispersed in absolute ethanol. For all nanoparticles, the reaction with PDI was performed, simultaneously, in exactly the same conditions, during 48 hours. The amount of PDI at the particles surface was calculated from the absorption spectra of the SiNPs, by subtracting the light scattering contribution (Fig. S9, Supporting Information). The amount of PDI obtained, 0.1 molecules/nm^2^, corresponds to 283, 342 and 580 PDI molecules per particles for GNPSi7, GNPSi9 and GNPSi13.

#### *Colloidal concentrations*

To calculate the concentration of gold in the dispersions we considered that all the gold is reduced to form nanoparticles, and use the diameter of the gold nanoparticles obtained by TEM to calculate their number concentration. All core-shell particles were prepared simultaneously from the same gold nanoparticles, and then also simultaneously processed. We verified that no particles were present in the supernatants after each centrifugation. The concentration of particles in the dispersions used for spectra measurements were compared with the concentration obtained from the Nanoparticle Tracking Analysis (NTA) measurements, to verify that the number concentration of particles is in fact very similar for all samples (Fig. S10, Supporting Information). The hydrodynamic diameter distributions measured by NTA, a light scattering based technique providing direct number-averaged diameter distributions, correspond to larger average diameter values than those obtained by TEM. A relatively small fraction of aggregates is observed in some samples, presumably due to flocculation in the dispersion, since TEM images show very few aggregates (Fig. 1 and Figs S6–S8, Supporting Information). Therefore, the hydrodynamic diameter of the particles is indeed much larger than the diameter obtained by TEM, probably because of the adsorbed PEG layer increasing the drag coefficient of the particles^57^. We can thus assume that the similar number concentrations obtained for all samples confirms our approach in calculating the colloidal concentrations.

### Characterization

The absorption spectra were recorded on a Shimadzu UV-3101PC UV-vis-NIR spectrophotometer and a Jasco V-660 spectrophotometer. The fluorescence measurements were obtained on a Horiba Jobin Yvon Fluorolog-3 spectrofluorometer.

TEM images were obtained on a Hitachi transmission electron microscope (Model H-8100 with a LaB6 filament) with an accelerator voltage of 200 kV. One drop of dispersion was placed on a carbon grid and dried in air before observation. The images obtained by TEM were binarized and analysed to measure the statistical nanoparticle diameter in order to obtain the particle size distribution curves.

Nanoparticle Tracking Analysis (NTA, Malvern Instruments) was used to determine the number-averaged hydrodynamic diameter distributions of the nanoparticles. Several runs of 90 s acquisitions were taken for each measurement, refreshing the fluid between each run. The results were collected in a histogram for better statistical significance.

Time-resolved picosecond fluorescence intensity decays were obtained by the single-photon timing method. The setup consists of a diode-pumped solid state Nd:YVO4 laser (Milennia Xs, Spectra Physics) synchronously pumping a mode-locked Ti:sapphire laser (Tsunami, Spectra Physics, with tuning range 700–1000 nm, output pulses of 100 fs, and 80 MHz repetition rate that can be reduced to 4 MHz by a pulse picker) or a cavity dumped dye laser (701-2, Coherent, delivering 3–4 ps pulses of *ca*. 40 nJ pulse-1 at 3.4 MHz) working with rhodamine 6G. Intensity decay measurements were made by alternating collection of impulse and decays with the emission polarizer set at the magic angle position. Impulses were recorded slightly away from the excitation wavelength with a scattering suspension. For the decays, a cutoff filter was used to effectively remove excitation light. Emission light was passed through a depolarizer before reaching the monochromator (Jobin-Yvon HR320 with a 100 lines/mm grating) and detected using a Hamamatsu 2809U-01 microchannel plate photomultiplier. No less than 10 000 counts were accumulated at the maximum channel. The decay curves were analysed using a nonlinear least squares re-convolution method.

## Electronic supplementary material

Supplementary Information

## References

[1] Halas NJ, Lal S, Chang W-S, Link S, Nordlander P (2011). Plasmons in strongly coupled metallic nanostructures. Chem. Rev..

[2] Jain PK, Huang X, El-Sayed IH, El-Sayed MA (2008). Noble metals on the nanoscale: optical and photothermal properties and some applications in imaging, sensing, biology, and medicine. Accounts of Chemical Research.

[3] Olson J (2015). Optical characterization of single plasmonic nanoparticles. Chem. Soc. Rev..

[4] Murphy CJ (2008). Gold nanoparticles in biology: beyond toxicity to cellular imaging. Accounts of Chemical Research.

[5] Linic S, Christopher P, Ingram DB (2011). Plasmonic-metal nanostructures for efficient conversion of solar to chemical energy. Nature Mater..

[6] Saha K, Agasti SS, Kim C, Li X, Rotello VM (2012). Gold nanoparticles in chemical and biological sensing. Chem. Rev..

[7] Zhao T (2014). Gold nanorod enhanced two-photon excitation fluorescence of photosensitizers for two-photon imaging and photodynamic therapy. ACS Appl. Mater. Interfaces.

[8] Pons T (2007). On the quenching of semiconductor quantum dot photoluminescence by proximal gold nanoparticles. Nano letters.

[9] Li X, Qian J, Jiang L, He S (2009). Fluorescence quenching of quantum dots by gold nanorods and its application to DNA detection. Appl. Phys. Lett..

[10] Reineck P (2013). Distance and wavelength dependent quenching of molecular fluorescence by Au@SiO_2_ core-shell nanoparticles. ACS nano.

[11] Ribeiro T, Prazeres TJV, Moffitt M, Farinha JPS (2013). Enhanced photoluminescence from micellar assemblies of cadmium sulfide quantum dots and gold nanoparticles. J. Phys. Chem. C.

[12] Wang L, Song Q, Liu Q, He D, Ouyang J (2015). Plasmon-enhanced fluorescence-based core–shell gold nanorods as a near-IR fluorescent turn-on sensor for the highly sensitive detection of pyrophosphate in aqueous solution. Adv. Funct. Mater..

[13] Lin H-H, Chen I-C (2015). Study of the interaction between gold nanoparticles and rose bengal fluorophores with silica spacers by time-resolved fluorescence spectroscopy. J. Phys. Chem. C.

[14] Ren Z (2015). Solution-based metal enhanced fluorescence with gold and gold/silver core–shell nanorods. Optics Communications.

[15] Reboud V (2013). Metallic nanoparticles enhanced the spontaneous emission of semiconductor nanocrystals embedded in nanoimprinted photonic crystals. Nanoscale.

[16] Fu Y, Zhang J, Lakowicz JR (2007). Plasmonic enhancement of single-molecule fluorescence near a Silver Nanoparticle. Journal of Fluorescence.

[17] Cheng, D. & Xu, Q.-H. Separation distance dependent fluorescence enhancement of fluorescein isothiocyanate by silver nanoparticles. *Chem*. *Commun*. 248–250 (2007). 10.1039/b612401a 17299628

[18] Chen J, Jin Y, Fahruddin N, Zhao JX (2013). Development of gold nanoparticle-enhanced fluorescent nanocomposites. Langmuir.

[19] Cheng Y (2011). Fluorescence near gold nanoparticles for DNA sensing. Analytical chemistry.

[20] Kochuveedu ST, Kim DH (2014). Surface plasmon resonance mediated photoluminescence properties of nanostructured multicomponent fluorophore systems. Nanoscale.

[21] Anger P, Bharadwaj P, Novotny L (2006). Enhancement and quenching of single-molecule fluorescence. Phys. Rev. Lett..

[22] Abadeer NS, Brennan MR, Wilson WL, Murphy CJ (2014). Distance and plasmon wavelength dependent fluorescence of molecules bound to silica-coated gold nanorods. ACS nano.

[23] Cang H (2011). Probing the electromagnetic field of a 15-nanometre hotspot by single molecule imaging. Nature.

[24] Razgoniaeva N (2015). Exciton generation in semiconductor nanocrystals via the near-field plasmon energy transfer. J. Phys. Chem. C.

[25] Darvill D, Centeno A, Xie F (2013). Plasmonic fluorescence enhancement by metal nanostructures: shaping the future of bionanotechnology. Phys. Chem. Chem. Phys..

[26] Khatua S (2014). Resonant plasmonic enhancement of single-molecule fluorescence by individual gold nanorods. ACS nano.

[27] Vukovic S, Corni S, Mennucci B (2009). Fluorescence enhancement of chromophores close to metal nanoparticles. Optimal setup revealed by the polarizable continuum model. J. Phys. Chem. C.

[28] Noginov MA (2009). Demonstration of a spaser-based nanolaser. Nature.

[29] Yuan H, Khatua S, Zijlstra P, Yorulmaz M, Orrit M (2013). Thousand-fold enhancement of single-molecule fluorescence near a single gold nanorod. Angew. Chem. Int. Ed..

[30] Li H (2012). Highly sensitive detection of proteins based on metal-enhanced fluorescence with novel silver nanostructures. Analytical Chemistry.

[31] Wientjes E, Renger J, Curto AG, Cogdell R, Hulst NFV (2014). Strong antenna-enhanced fluorescence of a single light-harvesting complex shows photon antibunching. Nature Comm..

[32] Naiki H (2013). Highly controlled plasmonic emission enhancement from metal-semiconductor quantum dot complex nanostructures. J. Phys. Chem. C.

[33] Chatterjee S, Lee JB, Valappil NV, Luo D, Menon VM (2011). Investigating the distance limit of a metal nanoparticle based spectroscopic ruler. Biomedical Optics Express.

[34] Zhang J (2012). pH- and glucose-responsive core-shell hybrid nanoparticles with controllable metal-enhanced fluorescence effects. ACS Appl. Mater. Int..

[35] Tang F, Ma N, Tong L, He F, Li L (2012). Control of metal-enhanced fluorescence with pH- and thermoresponsive hybrid microgels. Langmuir.

[36] Guerrero-Martínez A, Pérez-Juste J, Liz-Marzán LM (2010). Recent progress on silica coating of nanoparticles and related nanomaterials. Adv. Mater..

[37] Tam F, Goodrich GP, Johnson BR, Halas NJ (2007). Plasmonic enhancement of molecular fluorescence. Nano Letters.

[38] Ribeiro T, Baleizão C, Farinha JPS (2009). Synthesis and characterization of perylenediimide labeled core-shell hybrid silica-polymer nanoparticles. J. Phys. Chem. C.

[39] Santiago, A. M. *et al*. Multifunctional hybrid silica nanoparticles with a fluorescent core and active targeting shell for fluorescence imaging biodiagnostic applications. *Eur*. *J*. *Inorg*. *Chem*. 4579–87 (2015).

[40] Zhang D, Nettles CB (2015). A generalized model on the effects of nanoparticles on fluorophore fluorescence in solution. J. Phys. Chem. C.

[41] Prazeres TJV, Beija M, Charreyre M-T, Farinha JPS, Martinho JMG (2010). RAFT polymerization and self-assembly of thermoresponsive poly(N-decylacrylamide-b-N,N-diethylacrylamide) block copolymers bearing a phenanthrene fluorescent α-end group. Polymer.

[42] Rickard D, Giordani S, Blau WJ, Coleman JN (2008). Quantifying the contributions of inner-filter, re-absorption and aggregation effects in the photoluminescence of high-concentration conjugated polymer solutions. J. Luminescence.

[43] Conte JC, Martinho JMG (1981). Radiative energy transfer I. General equations. J. Luminescence.

[44] Turkevich J, Stevenson PC, Hillier J (1951). A study of the nucleation and growth processes in the synthesis of colloidal gold. Discuss. Faraday Soc..

[45] Liu S, Han M (2005). Synthesis, functionalization, and bioconjugation of monodisperse, silica-coated gold nanoparticles: robust bioprobes. Advanced Functional Materials.

[46] Fernández-López C (2009). Highly controlled silica coating of PEG-capped metal nanoparticles and preparation of SERS-encoded Particles. Langmuir.

[47] Szekeres M, Tóth J, Dékány I (2002). Specific surface area of stoeber silica determined by various experimental methods. Langmuir.

[48] Alvarez MM (1997). Optical absorption spectra of nanocrystal gold molecules. J. Phys. Chem. B.

[49] Rosa JP, Lima JC, Baptista PV (2011). Experimental photophysical characterization of fluorophores in the vicinity of gold nanoparticles. Nanotechnology.

[50] Weitz JDA, Garoff S, Gersten JI, Nitzan A (1983). The enhancement of Raman scattering, resonance Raman scattering, and fluorescence from molecules adsorbed on a rough silver surface. Chem. Phys..

[51] Martinho JMG, Prazeres TJV, Moura L, Farinha JPS (2009). Fluorescence of oligonucleotides adsorbed onto the thermoresponsive poly(isopropyl acrylamide) shell of polymer nanoparticles: Application to bioassays. Pure Appl. Chem..

[52] Schuller JA (2010). Plasmonics for extreme light concentration and manipulation. Nature Mat..

[53] Jain PK, El-Sayed MA (2008). Noble metal nanoparticle pairs: effect of medium for enhanced nanosensing. Nano Letters.

[54] Wertz E, Isaacoff BP, Flynn JD, Biteen JS (2015). Single-molecule super-resolution microscopy reveals how light couples to a plasmonic nanoantenna on the nanometer scale. Nano letters.

[55] Pustovit VN, Shahbazyan TV (2010). Plasmon-mediated superradiance near metal nanostructures. Phys. Rev. B.

[56] Stöber W, Fink A, Bohn E (1968). Controlled growth of monodisperse silica spheres in the micron size range. J. Colloid Interface Sci..

[57] Hill RJ, Li F (2013). Hydrodynamic drag coefficient for soft core–shell nanoparticles in hydrogels. Chemical Engineering Science.

