# Supplementary material for: Artefact-free Evaluation of Metal Enhanced Fluorescence in Silica Coated Gold Nanoparticles
Source: Sci Rep. 2017 May 26;7:2440. doi: 10.1038/s41598-017-02678-0 (PMC5446421; doi:10.1038/s41598-017-02678-0)
Supplement: Supplementary file 1 — Supplementary Information [file 41598_2017_2678_MOESM1_ESM.pdf]

Artefact-free Evaluation of Metal Enhanced Fluorescence in Silica Coated Gold Nanoparticles

Tânia Ribeiro, Carlos Baleizão and José Paulo S. Farinha*

Centro de Química-Física Molecular and Institute of Nanoscience and Nanotechnology, Instituto Superior Técnico - Universidade de Lisboa, Av. Rovisco Pais 1049-001 Lisboa, Portugal.

** Corresponding author: farinha@tecnico.ulisboa.pt*

SUPPORTING INFORMATION

S1. Calculation of the extinction cross section of gold by the Mie theory

The extinction cross section (σ_{ext}) of the gold nanoparticles (GNP) can be calculated by the Mie theory using eq.S1.[1]

$$\sigma_{ext} = 9\varepsilon_m^{3/2} V \frac{\omega}{c} \frac{\varepsilon_2(\omega)}{[\varepsilon_1(\omega) + 2\varepsilon_m]^2 + \varepsilon_2^2(\omega)} \quad \text{(S1)}$$

In this expression, V is the spherical particle volume, c the speed of light, ω the angular frequency of the exciting radiation, ε_m is the dielectric constant of the surrounding medium, $\varepsilon_1(\omega)$ and $\varepsilon_2(\omega)$ denote the real and imaginary part of the dielectric function of the particle material, $\varepsilon_1(\omega) + i\varepsilon_2(\omega)$.

The optical constants of gold obtained by Johnson and Christy [2] are used to determine $\varepsilon_1(\omega)$ and $\varepsilon_2(\omega)$, however the contribution of the size is not taken into account. The first step in obtaining a size dependence for metal particles is the decomposition of the dielectric function into two terms, an interband contribution (*IB*), accounting for the response of 5d electrons and a free-electron contribution (also called the Drude contribution, *D*) from the electrodynamics of the nearly free conduction electrons [1]

$$\varepsilon_1(\omega) = \varepsilon_{1IB}(\omega) + \varepsilon_{1D}(\omega) \tag{S2}$$

$$\varepsilon_2(\omega) = \varepsilon_{2IB}(\omega) + \varepsilon_{2D}(\omega) \tag{S3}$$

$$\varepsilon_{1D}(\omega) = 1 - \frac{\omega_p^2}{\omega^2 + \Gamma^2} \tag{S4}$$

$$\varepsilon_{2D}(\omega) = \frac{\omega_p^2 \Gamma}{\omega(\omega^2 + \Gamma^2)} \tag{S5}$$

In these expressions, ω_p is the frequency of the plasma oscillation of free electrons (1.38x10^{16} s^{-1} for gold), [3] Γ is the frequency of inelastic collisions of free electrons within the metal which is given by the inverse of the relaxation time τ (τ = 9.30x10^{-15} s for gold). [3] To calculate the terms of the *IB* contribution, $\varepsilon_{1IB}(\omega)$ and $\varepsilon_{2IB}(\omega)$, we subtract $\varepsilon_1(\omega)$ and $\varepsilon_2(\omega)$ to $\varepsilon_{1D}(\omega)$ and $\varepsilon_{2D}(\omega)$, respectively (eqs. S2, S3).

The size dependence is introduced by assuming that as the size of the particle decreases, the rate of scattering from the particle surface (ω_S) begins to exceed the bulk scattering rate Γ. The surface scattering rate is expressed in terms of the Fermi velocity (υ_F = 1.39x10^6 m/s for gold) and particle radius *r*

$$\omega_s = \Gamma + \frac{A\upsilon_F}{r} \tag{S6}$$

This expression can be interpreted as a limitation on the mean free path of the free electrons by the particle dimensions. The proportionality factor *A* equals 1 for isotropic scattering.[1] To obtain the dielectric functions of the particles, it is further assumed that the *IB* contribution is unchanged from the bulk, but that free-electron contributions for small particles use ω_S in place of Γ in eqs S4 and S5. Using these total dielectric functions in equation S1, we obtain the theoretical extinction spectrum for GNPs with 15 nm diameter, which agrees with that obtained experimentally for GNPs in water (both normalized, Figure 1B).

S2. Calculation of expected electric field enhancement for GNPSi-PDI structures

Based on a dipolar coupling model,[4] the electric dipole moment μ of an isolated nanoparticle in an electric field E is given as

$$\mu = \alpha \varepsilon_m E \tag{S7}$$

where α is the Clausius-Mossotti dipole polarizability for an isolated metal nanoparticle in a quasi-static approximation, which for a sphere is expressed by

$$\alpha = 3\varepsilon_0 V \left(\frac{\varepsilon - \varepsilon_m}{\varepsilon + 2\varepsilon_m} \right) \tag{S8}$$

where V is the particle volume, ε is the wavelength-dependent dielectric constant and ε_m is the dielectric constant of the surrounding medium. If we substitute α in eq. S7, we obtain

$$\mu = 3\varepsilon_0 V \varepsilon_m E \left(\frac{\varepsilon - \varepsilon_m}{\varepsilon + 2\varepsilon_m} \right) \tag{S9}$$

In the presence of a metal nanoparticle, the electric field E exciting the chromophore is the sum of the incident light field E_{inc} and the near-field of the metal NP electric dipole. In the dipole limit, the near-field decays as the cube of the distance from the surface particle. If the light is polarized along the interparticle axis, we have [4]

$$E = E_{inc} + \frac{\mu}{2\pi \varepsilon_m \varepsilon_0 d^3} \tag{S10}$$

In our system the electric field at the PDI position in the silica surface (E_{PDI}) is thus

$$E_{PDI} = E_{inc} + \frac{\mu_{Au}}{2\pi \varepsilon_m \varepsilon_0 d^3} \tag{S11}$$

The electric field enhancement felt by the PDI in the presence of a GNP is then $\left(E_{PDI}/E_{inc}\right)^2$. If we consider no increase in the dye fluorescence quantum yield (as in the case of the PDI,

which have near unity quantum yield), the electric field enhancement is directly reflected on the dye emission, so that the emission amplification would be $EA = \left(E_{PDI}/E_{inc}\right)^2$.

In order to find the dependence of EA on the separation distance, we replace μ_{Au} in eq. S11 to obtain

$$EA = \left(1 + \frac{1}{d^3}\left(2r_{Au}{}^3\left(\frac{\varepsilon_{Au} - \varepsilon_m}{\varepsilon_{Au} + 2\varepsilon_m}\right)\right)\right)^2 \qquad \text{(S12)}$$

$$A = 2r_{Au}{}^3\left(\frac{\varepsilon_{Au} - \varepsilon_m}{\varepsilon_{Au} + 2\varepsilon_m}\right) \qquad \text{(S13)}$$

Replacing *A* in eq. S12, we obtained

$$EA = 1 + \frac{2A}{d^3} + \frac{A^2}{d^6} \qquad \text{(S14)}$$

The emission amplification for PDI is represented in Figure S5 as a function of the distance to the metal surface.

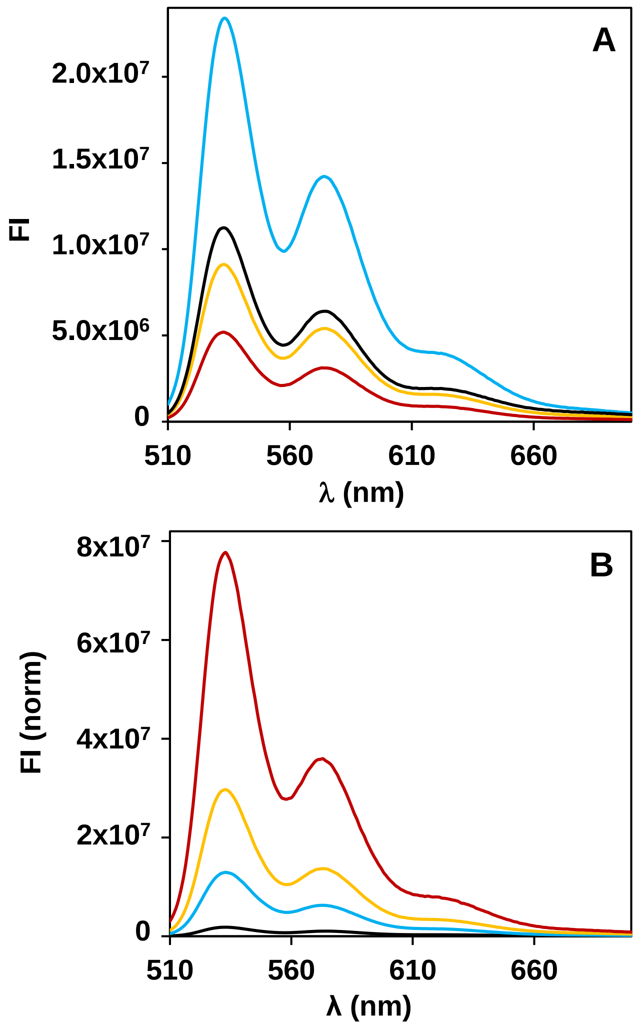

Figure S1. Emission spectra (λ_{ex}=480 nm, front-face acquisition in ethanol) obtained for SiNP-PDI (black curve), GNPSi13-PDI (blue curve), GNPSi9-PDI (yellow curve) and GNPSi7-PDI (red curve), before **(A)** and after **(B)** correction of the inner filter effects (eq. 1) and normalization at [PDI]=10^{-6} M.

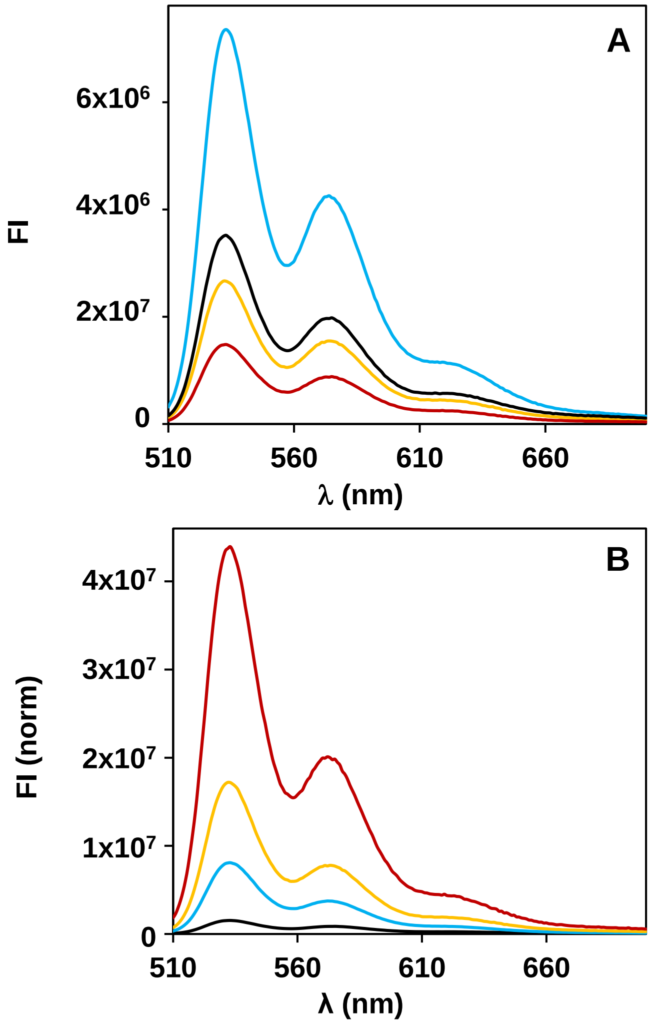

Figure S2. Emission spectra (λ_{ex}=480 nm, right-angle acquisition in ethanol, initial particle concentration diluted to 50%) obtained for SiNP-PDI (black curve), GNPSi13-PDI (blue curve), GNPSi9-PDI (yellow curve) and GNPSi7-PDI (red curve), before **(A)** and after **(B)** correction of the inner filter effects (eq. 1) and normalization at [PDI]=10^{-6} M.

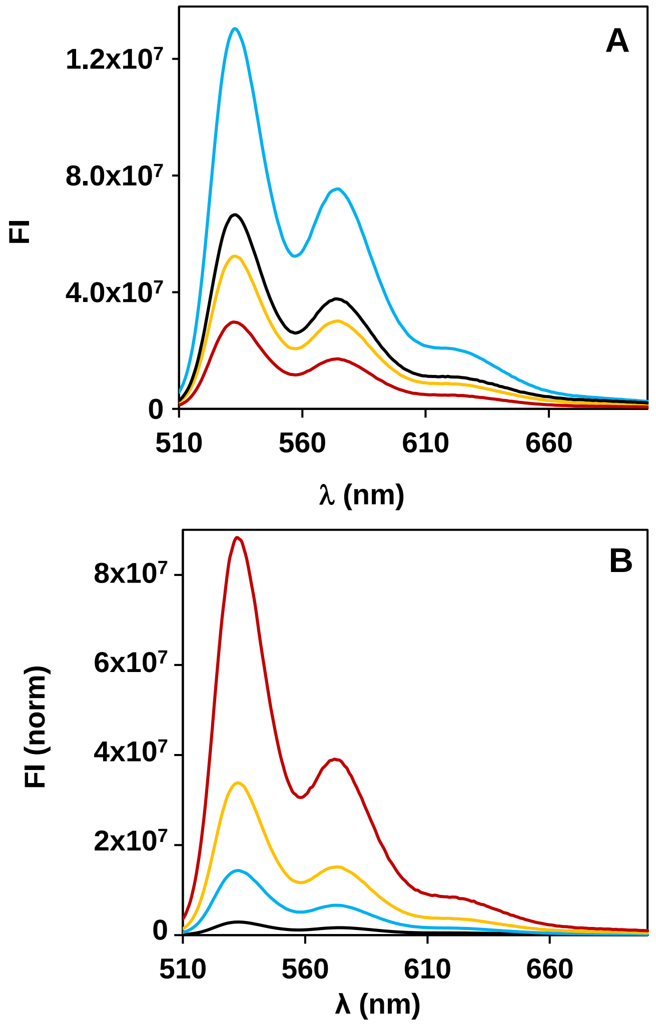

Figure S3. Emission spectra (λ_{ex}=480 nm, front-face acquisition in ethanol, initial particle concentration diluted to 50%) obtained for SiNP-PDI (black curve), GNPSi13-PDI (blue curve), GNPSi9-PDI (yellow curve) and GNPSi7-PDI (red curve), before **(A)** and after **(B)** correction of the inner filter effects (eq. 1) and normalization at [PDI]=10^{-6} M.

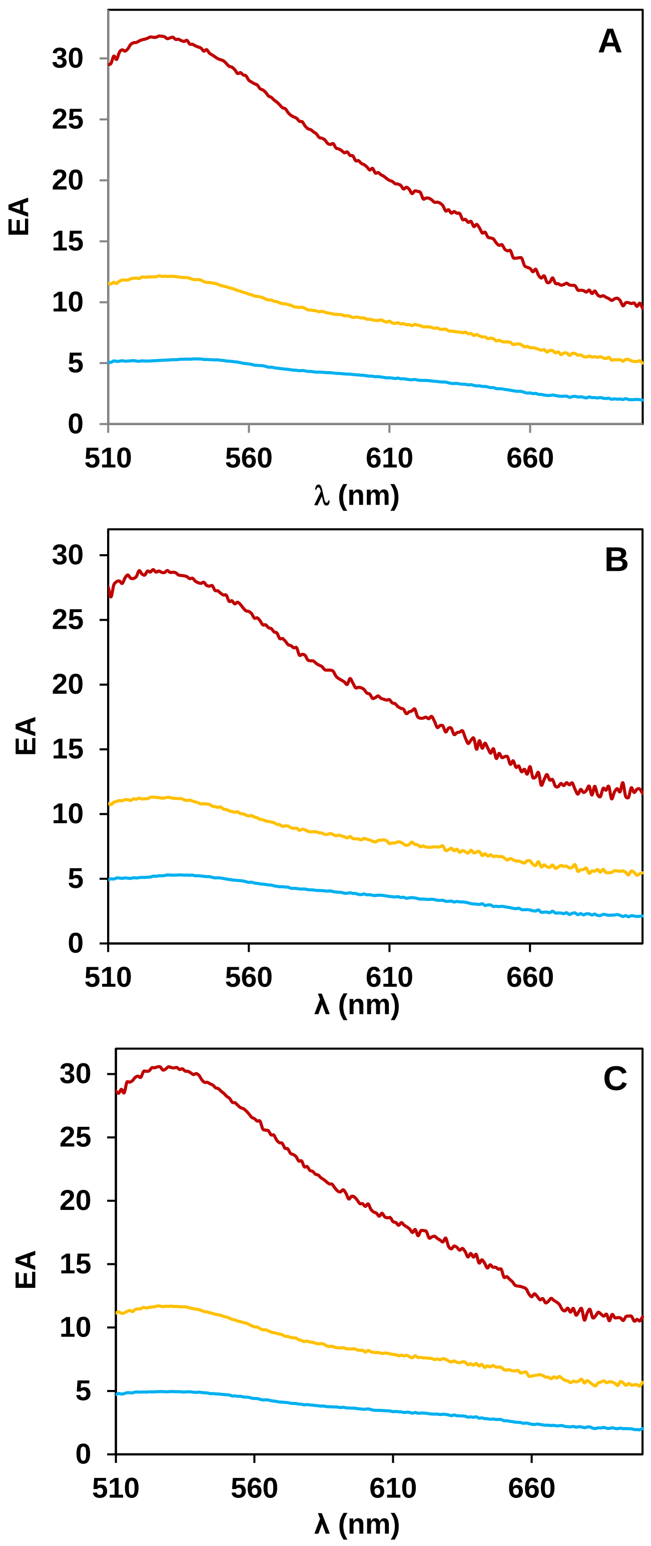

Figure S4. (A) The emission intensity amplification of GNPSi13-PDI (blue curve), GNPSi9-PDI (yellow curve) and GNPSi7-PDI (red curve), measured in front-face geometry. Emission intensity amplification of the same samples by diluting the initial concentration to 50%, measuring on right angle **(B)** and front-face **(C)** positions. The emission intensity amplification curves have maxima around the GNPs LSPR wavelength.

Table S1. Values of maximum amplification in the emission intensity (EA_{max}) for GNPSi13-PDI, GNPSi9-PDI and GNPSi7-PDI (and the same samples diluted to 50%), measuring at right angle and front-face geometries, and values of an average of EA_{max} for all samples.

	EA_{max}				
	[Au]	[Au] / 2	[Au]	[Au] / 2	
Detection	Right angle	Right angle	Front-face	Front-face	**Average EA_{max}**
GNPSi13-PDI	7	5	5	5	**6**
GNPSi9-PDI	13	11	12	12	**12**
GNPSi7-PDI	32	29	32	31	**31**

Table S2. Luminescence decay lifetimes obtained for the ethanol dispersions of the PDI, the GNPSi-PDI nanoparticles and SiNP-PDI.

	τ (ns)
GNPSi7-PDI	4.06
GNPSi9-PDI	4.09
GNPSi13-PDI	4.10
SiNP-PDI	4.09
PDI	4.13

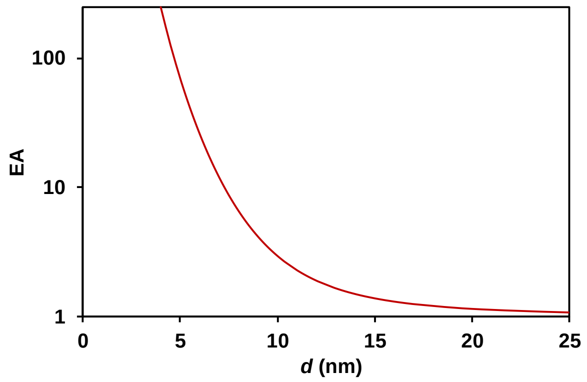

Figure S5. Representation of expected emission amplification (EA) of PDI as a function of the dye-metal separation distance *d*.

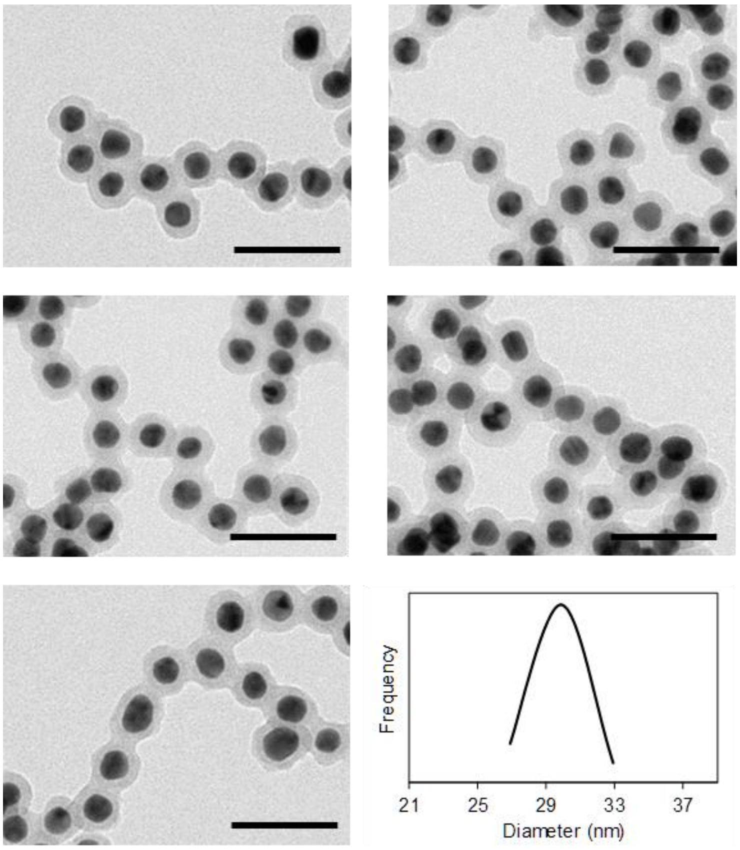

Figure S6. TEM images of GNPSi7 nanoparticles and diameter distribution curve calculated for close to 100 nanoparticles from several images (100 nm scale bars).

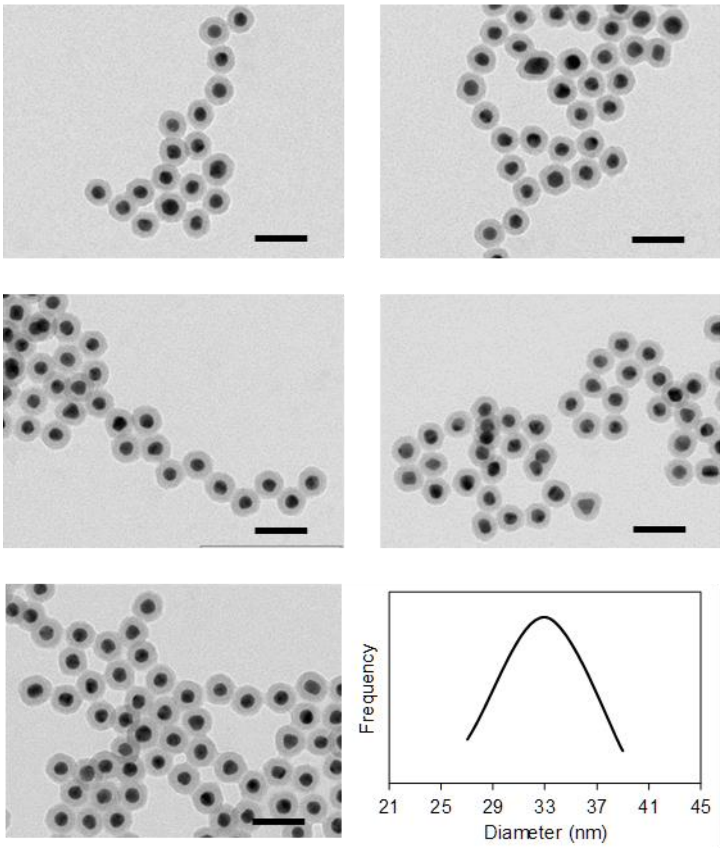

Figure S7. TEM images of GNPSi9 nanoparticles and diameter distribution curve calculated for close to 100 nanoparticles from several images (100 nm scale bars).

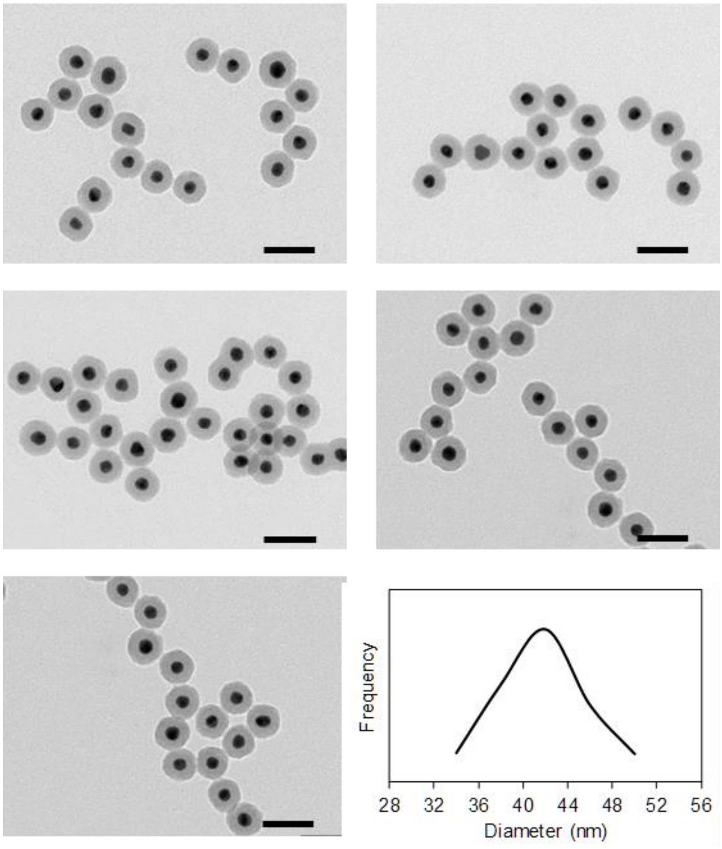

Figure S8. TEM images of GNPSi13 nanoparticles and diameter distribution curve calculated for close to 100 nanoparticles from several images (100 nm scale bars).

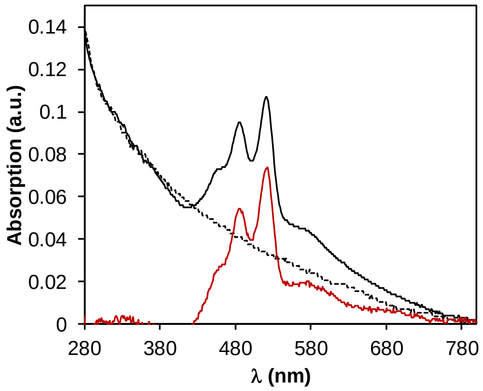

Figure S9. The amount of PDI at the SiNP-PDI surface was calculated from the absorption spectrum represented in red, obtained by subtracting the light scattering contribution (measured for the unlabeled SiNPs, dashed curve) from the absorption spectrum of SiNP-PDI (black solid curve). The scattering spectrum of the SiNP (dashed curve) was matched to the wavelength region of the SiNP-PDI where PDI does not absorb and thus, only scattering contributes to the spectra (below *ca.* 380 nm and above *ca.* 750 nm).

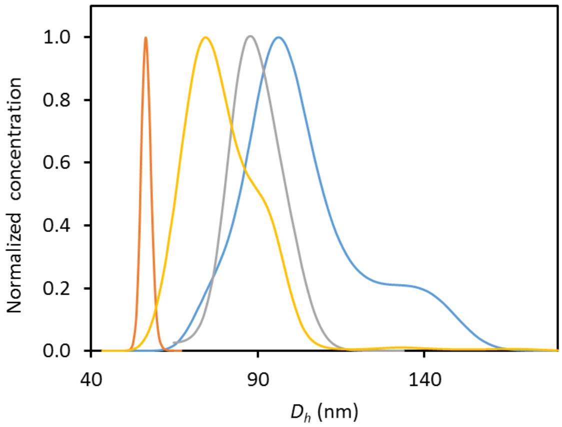

Figure S10. Hydrodynamic diameter distribution obtained by NTA of GNP (orange curve), GNPSi7-PDI (yellow curve), GNPSi9-PDI (grey curve) and GNPSi13-PDI (blue curve) nanoparticles, corresponding to average hydrodynamic diameters of (57±1) nm, (78±12) nm, (89±8) nm, and (101±18) nm, respectively. Mode or peak values of 56 nm, 88 nm, 74 nm and 96 nm, in the same order).

References

[1] Alvarez, M.M. *et al*. Optical Absorption Spectra of Nanocrystal Gold Molecules. *J. Phys. Chem. B* **101**, 3706-12 (1997).

[2] Johnson, P.B. & Christy, R.W. Optical Constants of the Noble Metals. *Phys. Rev. B* **6**, 4370-79 (1972).

[3] Kawata, S. Near-Field Optics and Surface Plasmon Polaritons. *Topics Appl. Phys.* **81**, 97-123 (2001).

[4] Jain, P.K. & El-Sayed, M.A. Noble Metal Nanoparticle Pairs: Effect of Medium for Enhanced Nanosensing. *Nano Letters* **8**, 4347-52 (2008).
